# Delayed maturation of the milk microbiome in women with type 1 diabetes

**DOI:** 10.1007/s00125-026-06791-6

**Published:** 2026-06-30

**Authors:** Alexandra J. Roth-Schulze, Katrina M. Ngui, Guinevere Martin, Pat Ashwood, Rebecca L. Thomson, Enrique Zozaya-Valdes, Yuan Gao, John M. Wentworth, Maria E. Craig, Anthony Huynh, Jennifer J. Couper, Megan A. S. Penno, Leonard C. Harrison

**Affiliations:** 1 Walter and Eliza Hall Institute of Medical Research, Melbourne, VIC Australia; 2 Department of Medical Biology, University of Melbourne, Melbourne, VIC Australia; 3 Adelaide University, Robinson Research Institute, Adelaide Medical School, University of Adelaide, Adelaide, SA Australia; 4 Department of Diabetes and Endocrinology, Royal Melbourne Hospital, Melbourne, VIC Australia; 5 School of Women’s and Children’s Health, Faculty of Medicine, University of New South Wales, Sydney, NSW Australia; 6 Institute of Endocrinology and Diabetes, The Children’s Hospital at Westmead, Sydney, NSW Australia; 7 Faculty of Health, Medicine and Behavioural Science, The University of Queensland, Brisbane, QLD Australia; 8 Department of Endocrinology & Diabetes, Queensland Children’s Hospital, South Brisbane, QLD Australia

**Keywords:** 16S rRNA, Breastmilk, Microbiome, Postpartum, Secretory IgA, Stool, Temporal, Trajectory, Type 1 diabetes

## Abstract

**Aims/hypothesis:**

The breastmilk microbiome plays a crucial role in gut microbial colonisation and immune development, but little is known about how it is influenced by type 1 diabetes.

**Methods:**

We conducted a longitudinal 16S rRNA gene sequencing study of milk from women with type 1 diabetes (*n*=69 pregnancies; 174 samples) and women who did not have type 1 diabetes (*n*=49 pregnancies; 123 samples), collected at seven timepoints from birth to 15 months postpartum. Alpha diversity (richness, inverse Simpson evenness) was analysed by generalised linear mixed models, beta diversity was analysed by Bray–Curtis dissimilarities and PERMANOVA, and differential abundance was analysed by limma. Additionally, we examined associations with maternal genetic risk score (GRS), maternal HLA type, glycaemic management (HbA_1c_) and breastmilk secretory IgA (sIgA), and performed a parallel analysis for the infant stool microbiome.

**Results:**

A significant interaction between type 1 diabetes status and timepoint was observed for alpha diversity, both richness (*p*=0.01) and inverse Simpson diversity (*p*=0.003), indicating distinct temporal trajectories between women with and without type 1 diabetes. In those without type 1 diabetes, richness increased significantly between birth and 1 week postpartum, but this early increase was delayed in women with type 1 diabetes to between 1 week and 3 months postpartum (*p*=0.002). Beta diversity analysis revealed earlier and more extensive compositional shifts in women without type 1 diabetes compared to those with type 1 diabetes. These differences persisted after adjusting for Caesarean delivery, BMI, parity and infant sex, and were not attributable to a delay in initiating breastfeeding. Taxa with delayed enrichment in women with type 1 diabetes included *Streptococcus* spp. and *Rothia mucilaginosa*, which metabolise human milk oligosaccharides to short-chain fatty acids to promote development of the infant’s gut barrier and immune system. Maternal GRS, HLA, HbA_1c_ or sIgA were not associated with milk microbiota diversity trajectories. In infant stool samples, alpha diversity did not differ between exposure groups, and showed no evidence of delayed maturation. Beta diversity revealed an early compositional shift between birth and 1 week postpartum only in infants born to women without type 1 diabetes. Similarly, significant taxonomic changes between birth and 1 week postpartum were detected only in infants born to women without type 1 diabetes, but with some taxa differing between exposure groups at 1 week.

**Conclusions/interpretation:**

Maternal type 1 diabetes is associated with delayed early maturation of the breastmilk microbiome. Early compositional differences in microbiota restructuring were also observed in the infant gut, partially mirroring the pattern in the milk microbiome; however, sustained differences in infant gut microbiota diversity were not detected. Further investigation could determine whether these changes affect development of the infant’s gut and immune system.

**Graphical Abstract:**

**Figure:**
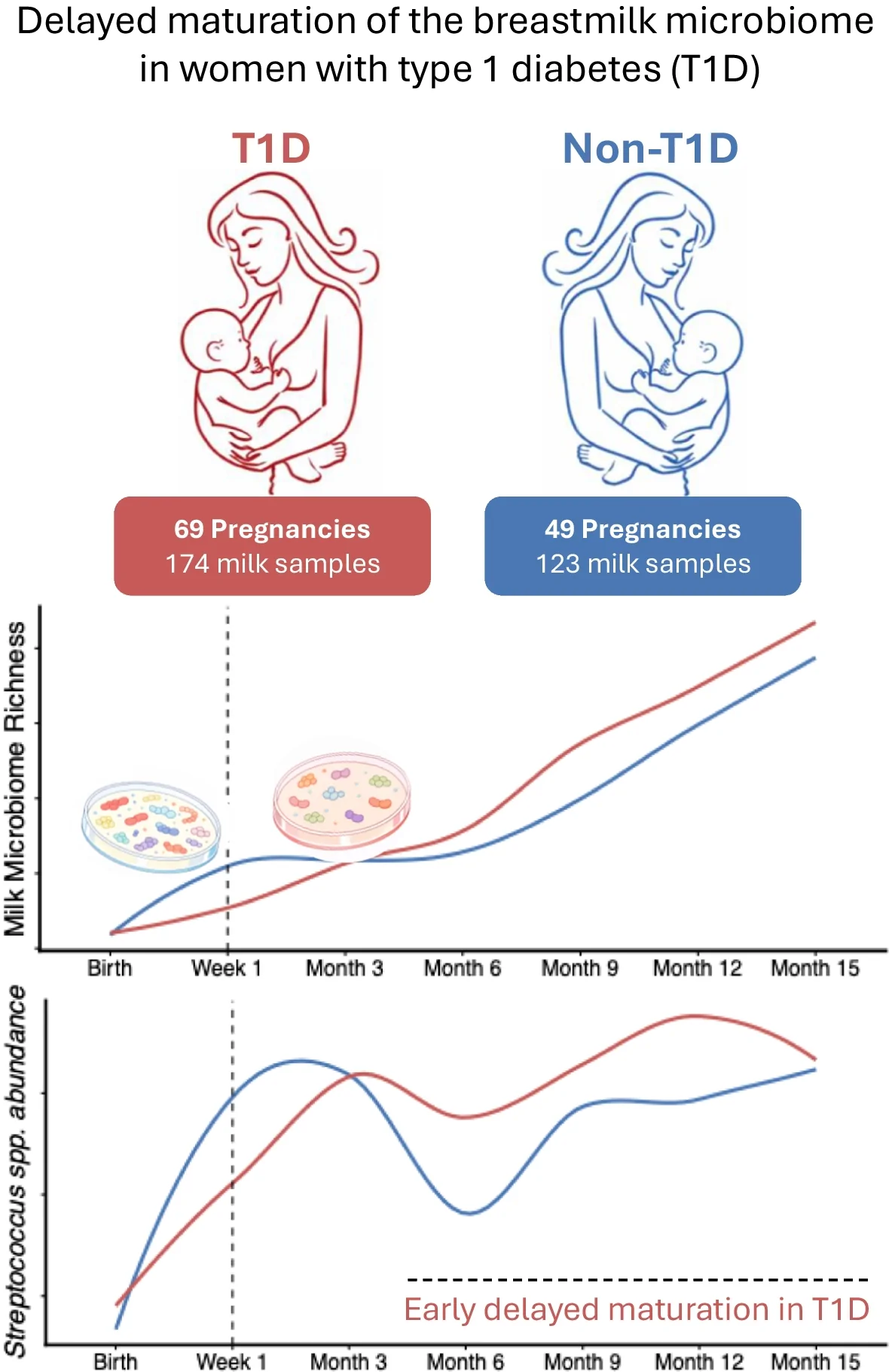

**Supplementary Information:**

The online version contains peer-reviewed but unedited supplementary material available at https://doi.org/10.1007/s00125-026-06791-6.

**Figure:**
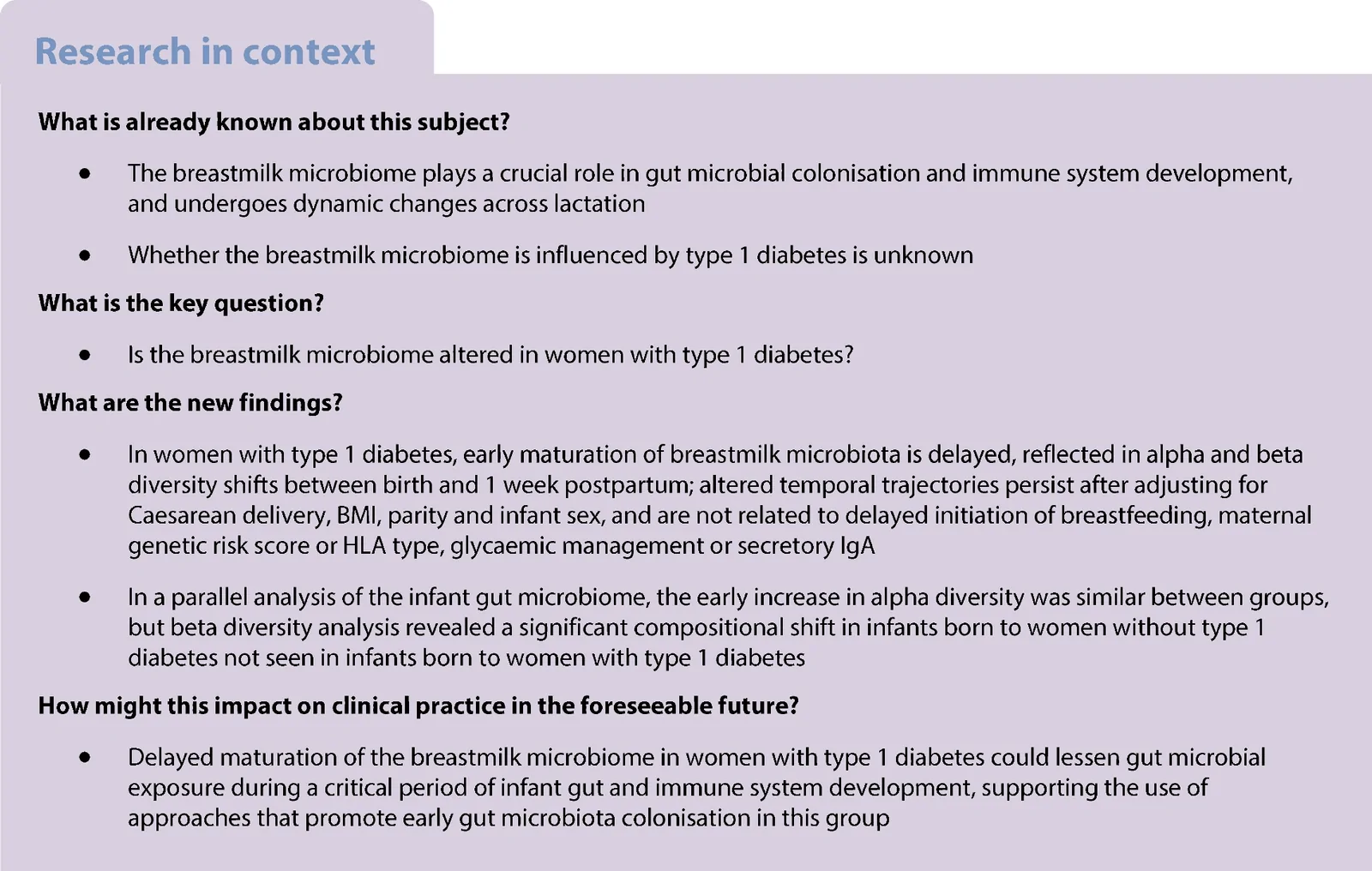

## Introduction

Human milk is a complex biological fluid that supports infant development by providing essential nutrients and a range of bioactive components, including immunoglobulins, hormones, oligosaccharides and microorganisms [1, 2]. The human milk microbiome, although low in biomass, is increasingly recognised as an important contributor to early-life gut microbial colonisation and to gut and immune development [3, 4]. It undergoes changes throughout lactation [5, 6] in distinct stages. Lactogenesis I, from mid-to-late pregnancy to 2–3 days postpartum, marks the onset of secretory differentiation and the accumulation of colostrum rich in immune components; lactogenesis II, from approximately 3 to 10–14 days postpartum, represents the onset of copious milk secretion accompanied by rapid shifts in milk composition [1, 7]; lactogenesis III refers to milk production maintained over the long term [8]. These stages, accompanied by gradual shifts in milk immune composition, from high concentrations of immunoglobulins and leukocytes to lower stable concentrations of secretory IgA (sIgA) and antimicrobial proteins, are critical ecological windows during which milk microbiota undergo succession, influenced by hormonal changes, nutrient availability and microbial transfer from maternal, infant and other sources [1, 5, 6, 9, 10].

Human milk harbours a low-biomass microbiome dominated by typical skin- and oral-associated bacteria [6, 10]. The most frequently reported milk phyla are Firmicutes, Proteobacteria, Actinobacteria and Bacteroidota [6, 10]. At the genus level, *Streptococcus* and *Staphylococcus* dominate, with relatively high abundance of *Rothia*, *Corynebacterium*, *Cutibacterium* (*Propionibacterium*), *Pseudomonas* and *Ralstonia* [6, 10–12]. A small subset of operational taxonomic units (OTUs) (approximately 20 taxa with ≥0.5% mean relative abundance) represents the majority of bacterial sequences [13]. Bacterial diversity tends to decline over the first 6 months postpartum [10]. Notably, *Bifidobacterium* and *Lactobacillus* peak in abundance around week 4 and then decline to ≤1%, but some genera, such as *Streptococcus*, *Staphylococcus*, *Pseudomonas*, *Acinetobacter*, *Bifidobacterium*, *Mesorhizobium*, *Brevundimonas*, *Flavobacterium* and *Rhodococcus*, may persist and fluctuate over time [10]. In mature milk, genera such as *Veillonella* become more prominent, reflecting progressive shifts in community composition [14].

Maternal health conditions may influence the composition and dynamics of milk microbiota. Gestational diabetes is associated with a change in the profile of human milk oligosaccharides [15] and hyperglycaemia, with decreased immunoglobulin concentrations in colostrum [16]. Type 1 diabetes has been shown to be associated with a delay in the onset of lactogenesis II [17]. However, the effect of type 1 diabetes on breastmilk composition, particularly on microbiota during the critical early postpartum period, is unexplored. To address this knowledge gap, we characterised breastmilk microbiome, by 16S rRNA gene amplicon sequencing, in women with and without type 1 diabetes, from birth to 15 months postpartum. We analysed changes in alpha and beta diversity, and differential abundance of taxa, in relation to lactation stage and maternal type 1 diabetes status, to determine whether type 1 diabetes is associated with altered microbial succession.

## Methods

### Study population

We analysed all available milk and infant stool samples collected between birth and 15 months postpartum from 118 pregnancies (69 in women with type 1 diabetes) enrolled in the ENDIA pregnancy–birth cohort study (www.anzctr.org.au, registration no. ACTRN12613000794707) [18]. Women were recruited at eight Australian sites between 2013 and 2018 and provided written informed consent. Ethics approval was granted by the Women’s and Children’s Health Network human research ethics committee (2020/HRE01400) under the National Mutual Acceptance Scheme, and by the Women and Newborn Health Service (RGS0000002639) and Child and Adolescent Health Service human research ethics committees (RGS0000002402) in Western Australia. Demographic, pregnancy and birth data were collected prospectively and verified from medical records (see electronic supplementary material [ESM] Methods: Study population).

### Sample collection and DNA extraction

Women self-collected milk at seven timepoints between birth and up to 15 months of age, termed B1 (0–3 days postpartum), B2 (4–10 days postpartum) and V1 to V5 for visits at three-monthly intervals from 3 months of age. Not all women provided samples at all timepoints. The mean time in days for sample collection was: B1, 0.9; B2, 6; V1, 98; V2, 186; V3, 278; V4, 375; V5, 452. Women hand-expressed milk with sterile gloves into a sterile container provided, and placed it in a refrigerator at 4°C before delivery to the laboratory within 24 h on ice for storage in liquid nitrogen. Samples were thawed on ice and centrifuged at 3000 *g* for 10 min at 4°C. The upper lipid layer was pierced with a pipette tip, and the whey infranatant removed using a fresh tip and stored at 4°C until assayed for sIgA within 3 days. The pellet was extracted for DNA using a Maxwell RSC PureFood pathogen kit (Promega). Women collected fresh infant stool samples using an EasySampler stool collection kit (ALPCO, Salem, NH, USA) at B1 after the first feed, at approximately 1 week of age (B2), and thereafter at approximately three-monthly visits coinciding with milk sampling, and stored them in a refrigerator at 4°C before delivery to the laboratory within 24 h on ice. Stool DNA was extracted using a MoBio PowerSoil kit (MoBio Laboratories, Carlsbad, CA, USA). DNA quality and quantity were assessed fluorometrically and spectrophotometrically before storage at −20°C until sequencing (see ESM Methods: Milk and infant stool sample collection and DNA extraction).

### DNA marker amplification and sequencing

The V4 region of the bacterial 16S rRNA gene was amplified using primers 515F/806R [19], and sequenced (2×300 bp) on a MiSeq sequencing system (Illumina). Each sample was amplified in duplicate, and sequencing runs included multiple negative controls (water, no-template and extraction blanks) to monitor contamination (see ESM Methods: DNA marker amplification and sequencing, and ESM Methods: Infant gut microbiome analysis).

### Bacterial sequence processing

Reads were processed independently per sequencing run using QIIME2 (version 2024.10) [20] using q2-dada2 plugin version 2024.10.0 [21] for de-noising and SILVA version 138 [22] for taxonomic classification. Run-specific processing allowed accurate error modelling and evaluation of batch effects. Tables of processed amplicon sequence variants (ASVs) and taxonomies were imported into R (version 4.4.2) [23] via the phyloseq package [24] (version 1.50.0) for integration, construction of a phylogenetic tree, and downstream analyses (see ESM Methods: Bacterial sequence analysis). Sequences classified as mitochondria, chloroplasts or unassigned were excluded, as were OTUs detected in negative control samples. Samples with insufficient reads after filtering were removed. This multistep curation minimised host-derived and reagent-associated contaminants (see ESM Methods: Removal of technical contaminants and non-bacterial sequences).

### Alpha diversity analysis

After rarefaction to 1000 reads, alpha diversity (richness and inverse Simpson index) was modelled using generalised linear mixed models (GLMMs) as implemented in the glmmTMB package [25] (version 1.1.10, accessed 18 April 2025) in R. Fixed covariates included type 1 diabetes status, timepoint, delivery mode, parity, infant sex and maternal BMI category. Model selection was guided by Akaike’s information criterion (AIC) to retain biologically relevant covariates. Model fit and assumptions were subsequently evaluated using residual diagnostics implemented in the DHARMa package [26] (version 0.4.7, accessed 18 April 2025), including assessment of residual patterns, dispersion and zero inflation. Post hoc pairwise comparisons of estimated marginal means (EMMs) were performed using the emmeans package version 1.11.1, accessed 20 April 2025 [27] in R, with *p* values corrected for multiple testing using a Benjamini–Hochberg false discovery rate (FDR) <0.05 (see ESM Methods: Alpha diversity analysis, and ESM Methods: Infant gut microbiome analysis).

### Beta diversity analysis

Microbial community composition was compared using Bray–Curtis dissimilarities and pairwise PERMANOVA (vegan version 2.7-1, accessed 30 May 2025) [28] at species to phylum levels. Analyses were conducted longitudinally (between consecutive timepoints) and cross-sectionally (type 1 diabetes vs non-type 1 diabetes at each timepoint), adjusting for sequencing run, participant ID, BMI category, delivery mode, infant sex and parity. Significance was defined as an FDR <0.05 (see ESM Methods: Beta diversity analysis, and ESM Methods: Infant gut microbiome analysis).

### Differential abundance analysis

Taxa differing between women with type 1 diabetes and those without type 1 diabetes were identified using limma [29] after trimmed mean of log expression ratios with singleton pairing (TMMwsp) normalisation [30] implemented in edgeR [31] and voom transformation [32, 33], adjusting for sequencing batch and maternal covariates. Contrasts tested group- and timepoint-specific effects, accounting for repeated measures via consensus correlation. The Benjamini–Hochberg procedure was used to control FDR. An FDR-adjusted *p* value <0.1, which is a common standard for statistical significance in exploratory analyses [34], was considered significant. Species-level identities of significant taxa were verified by BLASTn against the NCBI 16S ribosomal RNA database (accessed 20 June 2025) [35] (see ESM Methods: Differential abundance analysis, and ESM Methods: Infant gut microbiome analysis).

### Genetic risk score, genotyping for HLA and glycaemic management

The type 1 diabetes genetic risk score (GRS) [36] was analysed in the women without type 1 diabetes. The HLA-DR type, as previously determined [37], was included as a covariate in the primary longitudinal model. HbA_1c_ was measured in the women with type 1 diabetes. Associations with richness and inverse Simpson diversity were assessed using the same mixed-effects modelling framework described above (see ESM Methods: Alpha diversity analysis).

### Measurement and statistical analysis of sIgA

sIgA was measured in breastmilk whey samples by ELISA (E-EL-H1275 kit; Elabscience). Data were compared by Mann–Whitney *U* test using GraphPad Prism version 10.6.1, and correlations were assessed using Spearman’s rank correlation coefficient (cor.test) in R.

## Results

### Overview of milk microbiota composition

**Fig. 1:**
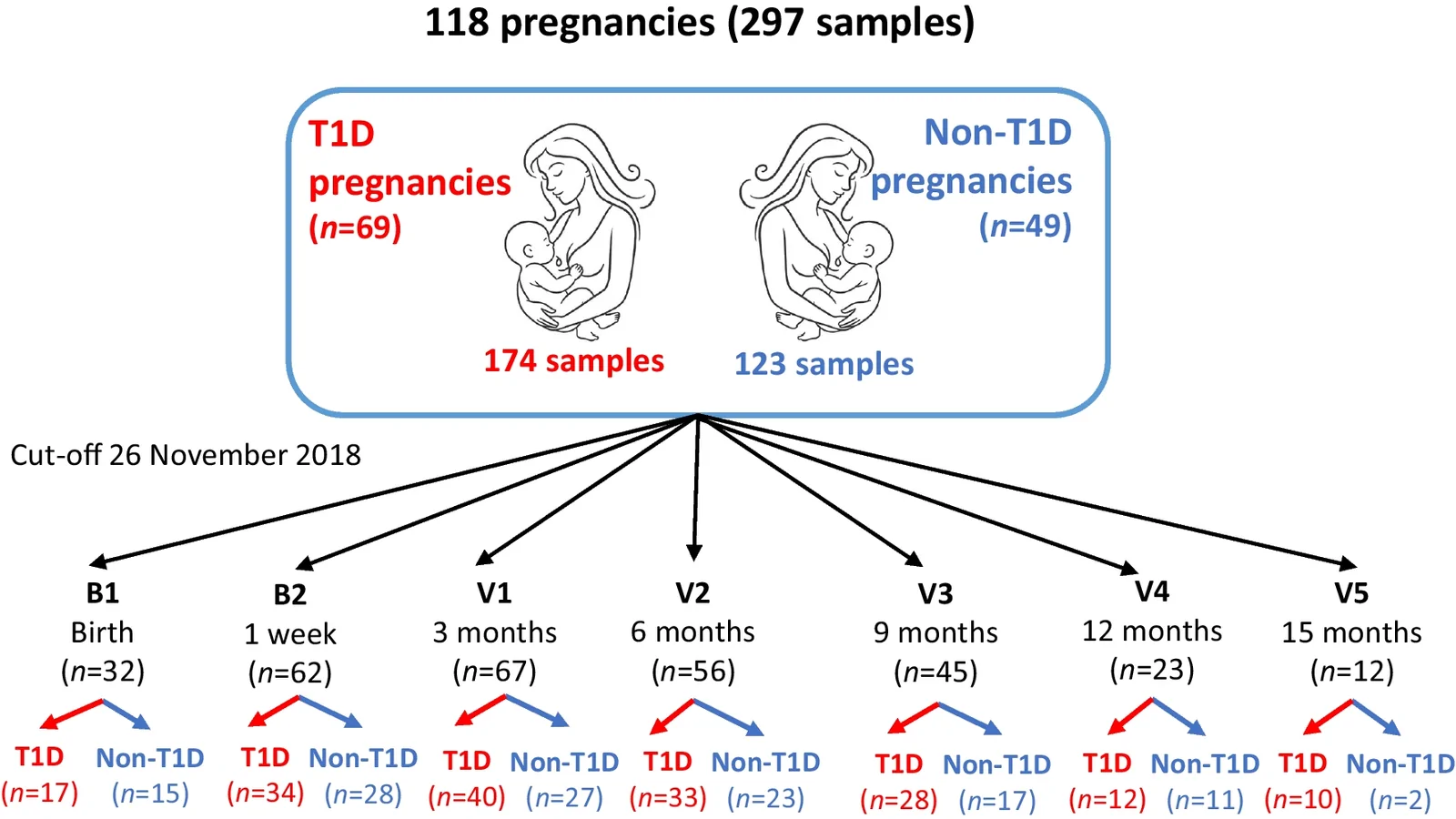
Numbers of breastmilk samples analysed longitudinally at seven timepoints (B1, 0–3 days postpartum (birth); B2, 4–10 days postpartum (1 week); V1, 3 months postpartum; V2, 6 months postpartum; V3, 9 months postpartum; V4, 12 months postpartum; V5, 15 months postpartum) in women with type 1 diabetes (T1D) and women without type 1 diabetes (non-T1D)

At the outset, a total of 427 milk samples from 138 pregnancies were available, including 247 from pregnancies in 81 women with type 1 diabetes pregnancies and 180 from pregnancies in 57 women without type 1 diabetes, spanning seven timepoints. After contaminant removal and filtering, 297 samples from 118 pregnancies remained, comprising 174 samples from pregnancies in 69 women with type 1 diabetes and 123 from pregnancies in 49 women without type 1 diabetes (Fig. 1). Participant characteristics are summarised in Table 1.

**Table 1:** Summary of characteristics of women with and without type 1 diabetes

	Women with type 1 diabetes (*n*=69)	Women without type 1 diabetes (*n*=49)	*p* value
Age at conception (years)			
Mean ± SD	32.1 ± 4.3)	33.2 ± 4.0	0.049*
Median [Q1, Q3]	31.4 [29.7, 34.9]	33.2 [30.8, 35.5]	
Birth country			1
Australia	57 (82.6)	40 (81.6)	
Overseas	12 (17.4)	9 (18.4)	
Pre-pregnancy BMI (kg/m^2^)			0.455
Underweight (<18.5)	2 (2.9)	0 (0.0)	
Healthy weight (18.5–24.9)	30 (43.5)	27 (55.1)	
Overweight (25.0–29.9)	21 (30.4)	10 (20.4)	
Obese (>30)	16 (23.2)	12 (24.5)	
HLA-DR^a^			0.01*
DR3,X; DR3,3	13 (18.8)	10 (20.4)	1
DR3,4	23 (33.3)	5 (10.2)	0.007**
DR4,X; DR4,4	24 (34.8)	20 (40.8)	0.57
DRX,X	8 (11.6)	14 (28.6)	0.026*
Undetermined	1 (1.4)	0 (0.0)	
HbA_1c_ (mmol/mol)			
Mean ± SD	46.5 ± 9.9	NM	
Median [Q1, Q3]	46.3 [40.0, 51.6]	NM	
Missing	1 (1.4)	NM	
HbA_1c_ (%)			
Mean ± SD	6.4 ± 0.9	NM	
Median [Q1, Q3]	6.4 [5.8, 6.9]	NM	
Missing	1 (1.4)	NM	
Parity			0.036*
0	38 (55.1)	16 (32.7)	
1	21 (30.4)	16 (32.7)	
>1	10 (14.5)	17 (34.7)	
Mode of birth^a^			0.00005***
Caesarean (elective)	26 (37.7)	9 (18.4)	0.026*
Caesarean (emergency)	22 (31.9)	5 (10.2)	0.0007***
Vaginal (assisted)	8 (11.6)	8 (16.3)	0.587
Vaginal (spontaneous)	13 (18.8)	27 (55.1)	0.0001***
Infant sex			0.58
Female	34 (49.3)	21 (42.9)	
Male	35 (50.7)	28 (57.1)	

Data are *n* (%) unless otherwise indicated

An unpaired *t* test was used for continuous variables and Fisher’s exact test was used for categorical variables (both two-sided)

^a^The multinomial Fisher’s exact test via Monte Carlo simulation was used for overall tests of categorical factors with more than two levels (4 × 2 Fisher/Monte Carlo test)

^*^p<0.05; **p<0.01; ***p<0.001

NM, not measured

After quality filtering, a median of 4713 paired-end bacterial reads per sample was obtained (range 1001–82,840). Overall, a total of 88 bacterial OTUs were identified: a median of 11 per sample (range 1–47). These OTUs were classified into eight phyla: 50.5% Firmicutes, 20.8% Actinobacteriota, 15.6% Bacteroidota, 9.8% Proteobacteria, 3.0% Fusobacteriota, 0.2% Campylobacterota, 0.09% Deinococcota and 0.05% Patescibacteria (ESM Fig. 1). Members of the genera *Streptococcus* (OTU1 and OTU2), *Rothia* (OTU3) and *Staphylococcus* (OTU4) were the dominant microbiota, comprising 20.0%, 12.7%, 11.2% and 8.8% of sequences, respectively (ESM Fig. 2). Temporal patterns were broadly similar between groups, but abundances differed, with *Streptococcus* (OTU1 and OTU2) and *Rothia* (OTU3) generally more abundant in women without type 1 diabetes during early lactation (ESM Fig. 3).

### Alpha diversity across timepoints in women with and without type 1 diabetes

**Fig. 2:**
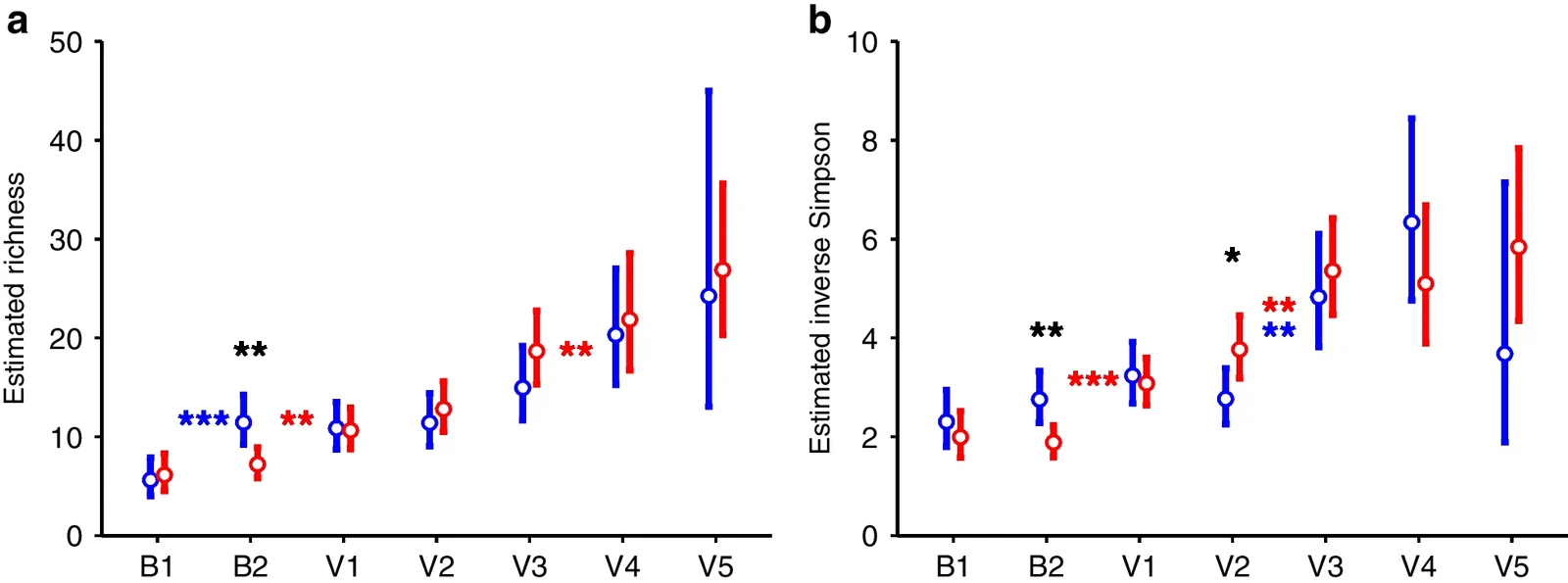
Temporal dynamics for alpha diversity of the milk microbiome in women with and without type 1 diabetes. Predicted means and 95% CI for (**a**) observed richness and (**b**) inverse Simpson alpha diversity indices across timepoints from birth (B1) and 1 week postpartum (B2) through to 15 months postpartum (V1–V5), derived from generalised linear mixed models with covariate adjustment. Blue, women without type 1 diabetes; red, women with type 1 diabetes. Asterisks indicate statistically significant pairwise differences between women with and without type 1 diabetes at the same timepoint (black) and between consecutive timepoints within the non-type 1 diabetes (blue) and type 1 diabetes (red) groups: *p<0.05; **p< 0.01; ***p<0.001 (FDR-adjusted)

A significant interaction between maternal type 1 diabetes status and timepoint was detected for both richness (*p*=0.01) and inverse Simpson diversity (*p*=0.003), indicating different temporal trajectories between groups. In women without type 1 diabetes, richness increased significantly between B1 and B2 (FDR-adjusted *p*=0.0004, contrast estimate=0.49; Table 2 and ESM Table 1), being 51% lower at B1 than at the B2 timepoint, with no significant changes across later timepoints (all *p*>0.07) (Fig. 2a, Table 2 and ESM Table 1). In contrast, women with type 1 diabetes showed no early increase in richness (B1 vs B2, *p*=0.35; Table 2 and ESM Table 1); instead, richness increased later, between B2 and V1 (*p*=0.002, contrast estimate=0.68) and between V2 and V3 (*p*=0.001, contrast estimate=0.69), indicating delayed maturation in women with type 1 diabetes (Fig. 2a, Table 2 and ESM Table 1).

**Table 2:** Breastmilk microbiome alpha diversity analysis: *p* values according to type 1 diabetes (T1D) status

Comparison	Adjusted *p* value
Richness	
Non-T1D	
B1 vs B2	0.0004***
B2 vs V1	0.74
V1 vs V2	0.74
V2 vs V3	0.09
V3 vs V4	0.09
V4 vs V5	0.70
T1D	
B1 vs B2	0.35
B2 vs V1	0.002**
V1 vs V2	0.10
V2 vs V3	0.001***
V3 vs V4	0.30
V4 vs V5	0.26
Non-T1D vs T1D	
B1	0.68
B2	0.001***
V1	0.88
V2	0.4
V3	0.13
V4	0.70
V5	0.76
Inverse Simpson	
Non-T1D	
B1 vs B2	0.32
B2 vs V1	0.32
V1 vs V2	0.32
V2 vs V3	0.001***
V3 vs V4	0.27
V4 vs V5	0.27
T1D	
B1 vs B2	0.73
B2 vs V1	0.00002***
V1 vs V2	0.08
V2 vs V3	0.005**
V3 vs V4	0.76
V4 vs V5	0.57
Non-T1D vs T1D	
B1	0.40
B2	0.003**
V1	0.69
V2	0.02*
V3	0.49
V4	0.28
V5	0.21

^*^p<0.05, **p<0.01, ***p<0.001

Inverse Simpson diversity, which captures both the number of taxa and the evenness of their distribution, did not mirror richness. In women without type 1 diabetes, community evenness increased only later in lactation (V2 to V3, *p*=0.001, contrast estimate=0.57), whereas it increased earlier in women with type 1 diabetes, between B2 and V1 *(p*=0.00002, contrast estimate=0.61) and again from V2 to V3 (*p*=0.005, contrast estimate=0.70) (Fig. 2b, Table 2 and ESM Table 1). Thus, trajectories differed by type 1 diabetes status, with a delayed increase in richness but an earlier increase in evenness in women with type 1 diabetes (Fig. 2b).

### Type 1 diabetes-associated differences in alpha diversity at individual timepoints

Pairwise comparisons were performed at each timepoint. Significant differences were observed only at B2 (lactogenesis II) (Fig. 2a, b, Table 2 and ESM Table 1). At this stage, richness was higher in women without type 1 diabetes compared to those with type 1 diabetes (*p*=0.001, contrast estimate=1.58, i.e. 58.4% higher). The InvSimpson index was also higher at B2 in women without type 1 diabetes (*p*=0.003, contrast estimate=1.46), but lower at V2 compared to women with type 1 diabetes (*p*=0.02, contrast estimate=0.74, i.e. 26.5% lower in women without type 1 diabetes) (Table 2 and ESM Table 1). No other differences were observed (all *p*>0.08; Table 2 and ESM Table 1).

### Roles of genetic risk, glycaemic management and sIgA

The GRS in non-type 1 diabetes women was not associated with richness or InvSimpson diversity, and did not modify temporal trajectories (ESM Table 2). HLA-DR type was included in the primary longitudinal model but did not attenuate the timepoint × type 1 diabetes interaction, and was not independently associated with diversity (ESM Table 2). In women with type 1 diabetes, HbA_1c_ was not associated with diversity or maturation dynamics (ESM Table 2).

**Fig. 3:**
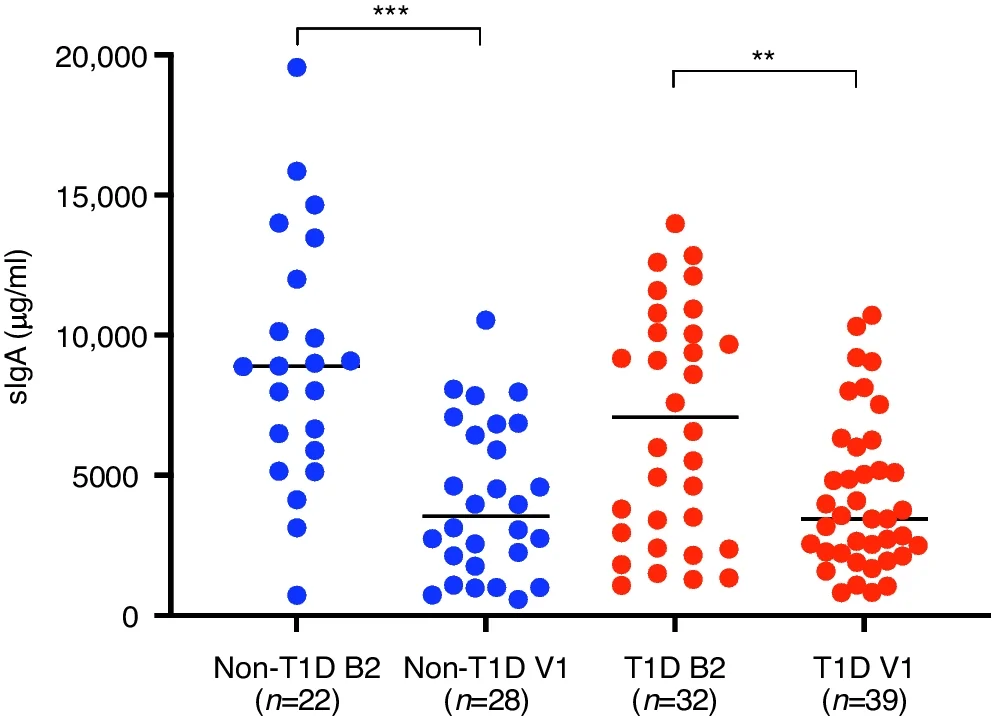
Breastmilk sIgA concentrations. sIgA was measured in women without type 1 diabetes (non-T1D) or with type 1 diabetes (T1D) at 1 week (B2) and 3 months (V1) postpartum. Horizontal lines are medians. *p* values are derived from Mann–Whitney two-sided *U* tests: ***p<0.001; **p<0.01

sIgA binds bacteria in the breastmilk and gut, and influences microbiome development [38]. Insufficient samples were available for analysis at B1. Between B2 and V1, sIgA decreased significantly in both women with type 1 diabetes and those without. However, sIgA did not differ between the groups at either B2 or V1 (Fig. 3). Spearman correlations between sIgA and alpha diversity at B2 (*n*=35) and V1 (*n*=48) showed no associations (B2 richness ρ=−0.21, *p=*0.23; B2 InvSimpson ρ=−0.23, *p*=0.19; V1 richness ρ=0.24, *p*=0.10; V1 InvSimpson ρ=0.1, *p*=0.51).

### Role of type 1 diabetes clinical factors in delayed lactogenesis II

Clinical indicators of delayed lactogenesis II, e.g. feeding of formula or expressed milk or infant separation due to placement in special care, were more frequent in women with type 1 diabetes. Overall, among women with non-missing data for this analysis, 84% (43/51) of those with type 1 diabetes had at least one such indicator, compared with 41% (16/39) of those without type 1 diabetes (Fisher’s exact test, two-tailed: OR 7.7, *p*=3.7 × 10^−5^) (ESM Table 3). Using a stricter definition (≤5 days breastfeeding in week 1) delayed lactogenesis II remained more common in women with type 1 diabetes (33%, 17/51) compared to women without type 1 diabetes (8%, 3/39) (*p*=0.005) (ESM Table 3). Caesarean section is a known risk factor for delayed lactogenesis II [39]. Among women with delayed lactation, Caesarean section was more frequent (76% with type 1 diabetes and 67% without) (ESM Table 3). As expected [40], Caesarean was more frequent in women with type 1 diabetes (69.6%) than in those without (28.6%) (*p*=0.00005; Table 1), and was therefore included as a covariate in diversity and differential abundance models.

### Beta diversity across timepoints in women with and without type 1 diabetes

**Fig. 4:**
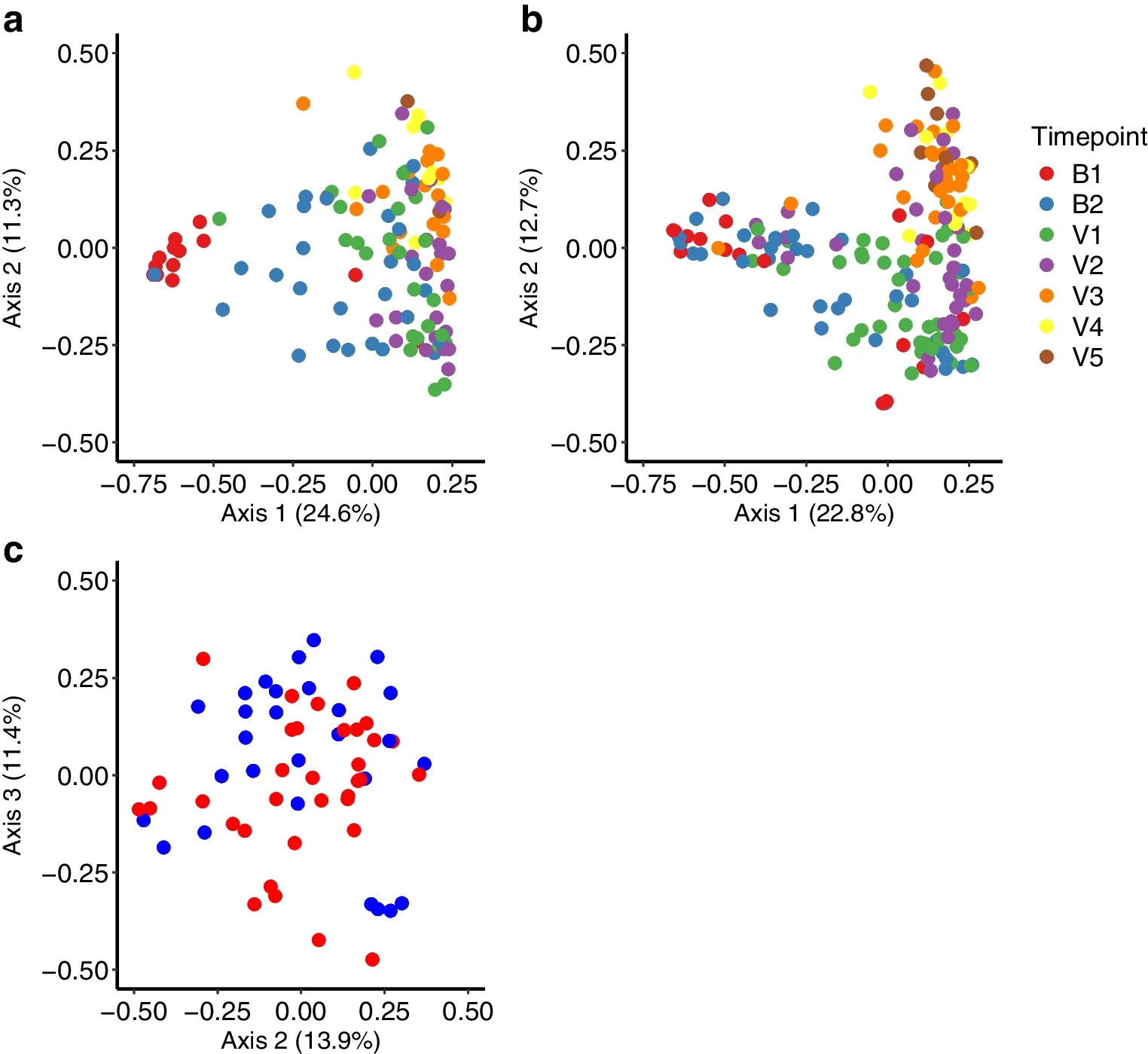
PCoA based on Bray–Curtis dissimilarities for temporal and type 1 diabetes-associated trends in milk microbiota composition at species taxonomic level. (**a**, **b**) PCoA plots showing the temporal progression of the milk microbiome composition in (**a**) women without type 1 diabetes and (**b**) women with type 1 diabetes. Each point represents a milk sample, coloured by lactation timepoint: B1, birth; B2, 1 week postpartum; V1, 3 months postpartum; V2, 6 months postpartum; V3, 9 months postpartum; V4, 12 months postpartum; V5, 15 months postpartum. Axes represent the first two principal coordinate dimensions, with the percentage of variance explained indicated in brackets. (**c**) PCoA analysis for women with type 1 diabetes (red) and without type 1 diabetes (blue) at the B2 timepoint; axes represent the second and third principal coordinate dimensions

We conducted pairwise PERMANOVA analyses of Bray–Curtis dissimilarities, adjusting for BMI category, parity, mode of delivery and infant sex. Significant differences in milk microbiota composition at the species level were observed between successive timepoints in women without type 1 diabetes. Significant shifts were observed between B1 and B2 (*p*_adjusted_=0.016, *R*^2^=5.8%), B2 and V1 (*p*_adjusted_=0.013, *R*^2^=4%), V1 and V2 (*p*_adjusted_=0.004, *R*^2^=5.6%) and V2 and V3 (*p*_adjusted_=0.006, *R*^2^=6.9%); differences were not detected beyond V3 (Table 3). In contrast, among women with type 1 diabetes, significant differences were found only between B2 and V1 (*p*_adjusted_=0.002, *R*^2^=4.1%), V1 and V2 (*p*_adjusted_=0.044, *R*^2^=2.6%) and V2 and V3 (*p*_adjusted_=0.006, *R*^2^=3.9%) (Table 3). The differences between B1 and B2 (*p*_adjusted_=0.581, *R*^2^=1.6%), and V3 vs V4 and V4 vs V5 were not significantly different (*p*_adjusted_>0.38; Table 3). Similar patterns were observed at other taxonomic levels (ESM Table 4). Consistently, principal coordinate analysis (PCoA) at the species level revealed a clear trajectory during lactation (from B1 to V5) in axis 1, explaining 24.6% of the variance in women without type 1 diabetes and 22.8% in those with type 1 diabetes (Fig. 4a, b). Axis 1 primarily reflects temporal changes in composition and was more compressed in women with type 1 diabetes.

**Table 3:** Breastmilk microbiome beta diversity analysis at species taxonomic level: *p* values according to type 1 diabetes (T1D) status

Comparison within levels	Adjusted *p* value
Non-T1D: B1 vs B2	0.016*
Non-T1D: B2 vs V1	0.013*
Non-T1D: V1 vs V2	0.004**
Non-T1D: V2 vs V3	0.006**
Non-T1D: V3 vs V4	0.108
Non-T1D: V4 vs V5	0.369
T1D: B1 vs B2	0.581
T1D: B2 vs V1	0.002**
T1D: V1 vs V2	0.044*
T1D: V2 vs V3	0.006**
T1D: V3 vs V4	0.898
T1D: V4 vs V5	0.384
B1: Non-T1D vs T1D	0.872
B2: Non-T1D vs T1D	0.042*
V1: Non-T1D vs T1D	0.872
V2: Non-T1D vs T1D	0.291
V3: Non-T1D vs T1D	0.788
V4: Non-T1D vs T1D	0.890
V5: Non-T1D vs T1D	0.630

^*^p<0.05, **p<0.01, ***p<0.001

### Type 1 diabetes-associated differences in beta diversity at individual timepoints

Comparison of the milk microbiome between women with and without type 1 diabetes at each timepoint, analysed by adonis2 (PERMANOVA with covariate adjustment), revealed significant compositional differences only at B2 (*p*_adjusted_=0.042, *R*^2^=3.6%; Table 3). Axis 3 separated women with and without type 1 diabetes at B2 (approximately 11.4% of the variance), indicating early postpartum compositional differences by type 1 diabetes status (Fig. 4c). No differences were observed at other timepoints (*p*_adjusted_>0.29; Table 3).

### Differentially abundant taxa across timepoints

**Fig. 5:**
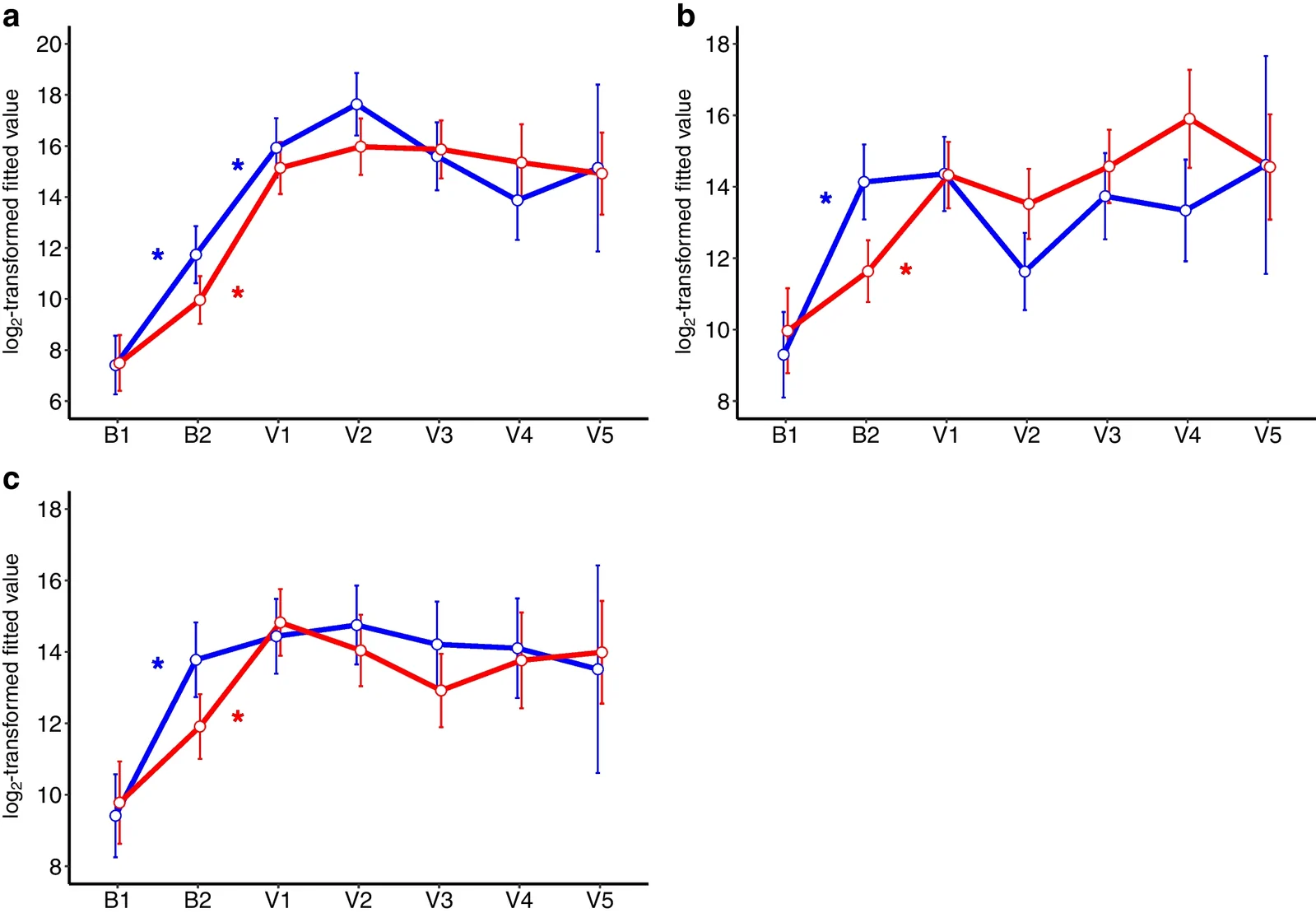
Differential abundance of key bacterial OTUs in human milk at various timepoints in women with and without type 1 diabetes, showing the abundance of (**a**) *Streptococcus* (OTU1), (**b**) *Streptococcus* (OTU2) and (**c**) *Rothia* (OTU3) in women without type 1 diabetes (blue) and women with type 1 diabetes (red). Values are means ± SEM. Asterisks indicate statistically significant differences between birth (B1) vs 1 week postpartum (B2) and between B2 and V1 (3 months) in women without type 1 diabetes and between B2 and V1 in women with type 1 diabetes (FDR-adjusted, *p*<0.1)

Differential abundance analysis revealed temporal shifts in milk microbiota composition aligned with beta diversity. Significant changes in OTU abundance occurred between B2 and V1, V1 and V2 and V2 and V3 in both groups, with taxa and timing differing by type 1 diabetes status. Notably, no taxa changed significantly between B1 and B2 in women with type 1 diabetes. In contrast, in women without type 1 diabetes, three OTUs were significantly enriched at B2 compared with B1 (FDR<0.009), two that were assigned to *Streptococcus* (OTU1: *p*=0.009, log_2_FC=−4.33; OTU2: *p*=0.005, log_2_FC=−4.84) and one assigned to the genus *Rothia* (OTU3: *p*=0.008, log_2_FC=−4.36) (Fig. 5). These three OTUs were the three most abundant in milk (20.0%, 12.7% and 11.2% of sequences, respectively) (ESM Fig. 2). The same OTUs increased later (between B2 and V1) in women with type 1 diabetes, indicating a delayed shift in community composition (Fig. 5, Table 4 and ESM Table 5). BLAST-based taxonomic refinement revealed that *Streptococcus* (OTU1) aligned with *S. infantis*, *S. lactarius*, *S. australis*, *S. oralis* and *S. ruberi*, and *Streptococcus* (OTU2) with *S. parasanguinis*, *S. rubneri*, *S. dentalis*, *S. lingualis*, *S. australis*, *S. oralis* and *S. koreensis*, all of which are members of the *Streptococcus mitis* group, a clade that is known for early maternal–infant microbial transmission and mucosal adaptation. *Rothia* (OTU3) exhibited 100% identity and coverage with *Rothia mucilaginosa*. Although the differences were not significant, the abundance profiles of these three OTUs suggest relative enrichment at B2 in women without type 1 diabetes compared to those with type 1 diabetes (Fig. 5, Table 4 and ESM Table 5).

**Table 4:** Differentially abundant bacterial taxa according to type 1 diabetes (T1D) status at the B2 timepoint and according to specific consecutive timepoints and group

Comparison	Group	Enriched	Taxa	log_2_FC^a^
B1 vs B2	Women without T1D	B2	*Streptococcus* (OTU1)	–4.33
B1 vs B2	Women without T1D	B2	*Streptococcus* (OTU2)	–4.84
B1 vs B2	Women without T1D	B2	*Rothia mucilaginosa* (OTU3)	–4.36
B2 vs V1	Women with T1D	V1	*Streptococcus* (OTU1)	–5.18
B2 vs V1	Women with T1D	V1	*Streptococcus* (OTU2)	–2.69
B2 vs V1	Women with T1D	V1	*Rothia mucilaginosa* (OTU3)	–2.91
B1 vs B2	Women without T1D	B2	Genus: *Streptococcus*	–5.22
B1 vs B2	Women without T1D	B2	*Genus: Rothia*	–3.30
B2 vs V1	Women with T1D	V1	*Genus: Streptococcus*	–4.22
B2 vs V1	Women with T1D	V1	*Genus: Rothia*	–2.51
B1 vs B2	Women without T1D	B2	Family: Micrococcaceae	–3.31
B1 vs B2	Women without T1D	B2	Family: Streptococcaceae	–5.51
B2 vs V1	Women with T1D	V1	Family: Micrococcaceae	–4.10
B2 vs V1	Women with T1D	V1	Family: Streptococcaceae	–2.37
B1 vs B2	Women without T1D	B2	Order: Lactobacillales	–4.41
B2 vs V1	Women with T1D	V1	Order: Lactobacillales	–4.27
T1D vs non-T1D	B2 timepoint only	Non-T1D	*Genus: Streptococcus*	3.00
T1D vs non-T1D	B2 timepoint only	Non-T1D	Family: Streptococcaceae	3.33
T1D vs non-T1D	B2 timepoint only	Non-T1D	Order: Lactobacillales	2.94
T1D vs non-T1D	B2 timepoint only	Non-T1D	Phylum: Fusobacteriota	2.77

^a^Values for the comparisons are log_2_ fold change

In addition, the genera *Streptococcus* (*p*=0.0002, log_2_FC=−5.22) and *Rothia* (*p*=0.084, log_2_FC=−3.30), the families Streptococcaceae (*p*=7 × 10^−5^, log_2_FC=−5.51) and Micrococcaceae (*p*=0.066, log_2_FC=−3.31), the order Lactobacillales (*p*=0.003, log_2_FC=−4.41) and the phyla Fusobacteriota (*p=*0.009, log_2_FC=−4.06), Campylobacterota (*p*=0.029, log_2_FC=−2.44) and Bacteroidota (*p*=0.08, log_2_FC=−3.6) were also significantly increased at B2 compared with B1 in women without type 1 diabetes, but not in women with type 1 diabetes (Table 4 and ESM Table 5). Notably, the same three OTUs, two genera and one order that were increased between B1 and B2 in women without type 1 diabetes also increased in women with type 1 diabetes but between B2 and V1 (Table 4 and ESM Table 5). Similarly, the three phyla that increased early in women without type 1 diabetes exhibited significant increases in women with type 1 diabetes only between V2 and V3 (ESM Table 5). No significant changes in beta diversity or differentially abundant OTUs were observed between V3 and V4 or between V4 and V5 in either group, except for the genus *Absconditabacteriales*_(SR1), which increased from V3 to V4 in women without type 1 diabetes (ESM Table 5). These results indicate delayed enrichment of key taxa in women with type 1 diabetes, with stabilisation in both groups from V3 onwards.

### Type 1 diabetes-associated differences in differential abundance at individual timepoints

At the 1 week postpartum timepoint (B2), no significant differences were detected at the OTU/species level between women with and without type 1 diabetes. However, significant differential abundance emerged at higher taxonomic levels. At the genus level, *Streptococcus* was significantly more abundant in women without type 1 diabetes (*p*=0.049, log_2_FC=3.00 Table 4). This enrichment was consistent at the family and order levels, with Streptococcaceae (*p*=0.013, log_2_FC=3.33) and Lactobacillales (*p*=0.033, log_2_FC=2.94) also being significantly more abundant in women without type 1 diabetes (Table 4 and ESM Table 5). The phylum Fusobacteriota was also enriched in women without type 1 diabetes (*p*=0.078, log_2_FC=2.77; Table 4 and ESM Table 5). These findings indicate that early compositional differences are more readily detected at higher taxonomic levels, with *Streptococcus* and related lineages being key discriminators at B2.

### Infant gut microbiome

The alpha diversity of the gut microbiome did not differ between infants born to women with type 1 diabetes and those born to women without type 1 diabetes at early timepoints, and did not show evidence of delayed maturation (ESM Table 6). In contrast, beta diversity analysis revealed a significant compositional shift between B1 and B2 in infants born to women without type 1 diabetes that was not detected in infants born to women with type 1 diabetes (ESM Table 7). No significant differences in overall community composition were observed between groups at B2 (ESM Table 7).

Differential abundance analysis identified 84 OTUs that were more abundant at B1 in infants born to women without type 1 diabetes (ESM Table 8). The majority (78) of these decreased in abundance from B1 to B2 in women without type 1 diabetes. Among these, five remained significantly more abundant at both B1 and B2 in infants born to women without type 1 diabetes (ESM Table 8). In contrast, infants born to women with type 1 diabetes maintained a low abundance of these taxa at both timepoints.

## Discussion

We show that development of the milk microbiome in the early postpartum period differs between women with and without type 1 diabetes. The taxonomy of the milk microbiome, which was dominated by a limited number of taxa, primarily skin and oral commensals such as *Streptococcus*, *Staphylococcus* and *Rothia* spp., is consistent with previous reports [6, 10, 41]. Differences were evident across alpha diversity, beta diversity and taxon-specific composition. Alpha diversity (richness and inverse Simpson index) was significantly lower in women with type 1 diabetes at 1 week postpartum (B2), indicating altered early microbial community establishment. In contrast, women without type 1 diabetes showed the expected increase in richness from birth (B1; 0–3 days postpartum) to 1 week (B2; 4–10 days postpartum), consistent with early microbial succession during the transition from colostrum to mature milk [5]. These findings identify B2 as a critical point at which the influence of maternal type 1 diabetes is most pronounced. This critical timepoint in lactogenesis II is marked by significant shifts in milk composition, including an increase in lactose and human milk oligosaccharides and a decline in immune components such as sIgA and lactoferrin that shape microbial growth [1]. The delay in microbiome maturation in women with type 1 diabetes was not explained by genetic risk of type 1 diabetes or glycaemic management, or by changes in sIgA, suggesting it is related to physiological changes associated with type 1 diabetes [15–17].

Consistent with previous studies [42], delayed lactogenesis II was more frequent among women with type 1 diabetes. Regardless of type 1 diabetes status, most women with delayed lactogenesis II delivered by Caesarean section, which is a known risk factor for a delay in milk ‘coming in’ [39] that could contribute to microbiota differences. Colostrum/milk was successfully collected at B1 and B2, and microbial differences were evident across these timepoints. Moreover, we adjusted for mode of delivery, making it unlikely that these differences are explained by Caesarean section-related delay.

The compositional differences in beta diversity between women with type 1 diabetes and those without further support delayed microbial succession in women with type 1 diabetes. In women without type 1 diabetes, significant shifts in microbial composition occurred across early timepoints, aligning with successive stages of lactation, but were delayed in women with type 1 diabetes. This was also reflected in differential abundance, whereby several OTUs, genera (*Streptococcus* and *Rothia*) and higher taxa increased between B1 and B2 in women without type 1 diabetes but not in those with type 1 diabetes. Strikingly, the three OTUs enriched at B2 compared with B1 in women without type 1 diabetes (*Streptococcus* OTU1, *Streptococcus* OTU2 and *Rothia* OTU3) were also the three most abundant OTUs in the overall community, together accounting for approximately 44% of all sequences. This overlap highlights the ecological relevance of these early shifts. At the B2 timepoint, *Streptococcus*, Streptococcaceae and Lactobacillales were significantly more abundant in women without type 1 diabetes, despite the absence of significant OTU/species-level differences. These lineage-wide patterns may reflect broader functional traits (e.g. metabolism of human milk oligosaccharides or mucosal adherence) that shape early colonisation. Notably, these taxa eventually increased in women with type 1 diabetes but not until B2/V1 or V2/V3, consistent with delayed maturation of their milk microbiome.

Prominent among the enriched early postpartum taxa in women without type 1 diabetes were members of the *Streptococcus* genus, which is known to dominate the milk microbiota [6, 10, 11], and *Rothia mucilaginosa*, all confirmed by ASV-level BLAST. These oral- and skin-associated commensals, which are frequently detected in the infant oral microbiome, are implicated in the retrograde flow of infant saliva into the mammary ducts during breastfeeding, facilitating microbial transmission between infant and breastmilk [6, 10]. They metabolise human milk oligosaccharides and mucins to produce short-chain fatty acids, which support gut barrier maturation, immune development and microbial homeostasis [43]. Their relative deficiency and delayed enrichment in breastmilk in women with type 1 diabetes could reduce early exposure to these metabolites during a critical window for gut and immune development.

To investigate whether these alterations translated into changes in the infant gut microbiome, we analysed longitudinal samples of stools from infants born to women in the study. Early community dynamics suggested a transient delay in microbial succession in infants born to women with type 1 diabetes. These differences resolved rapidly, indicating convergence during early infancy. These transient changes parallel those in the breastmilk microbiome, but the trajectories did not directly mirror each other, highlighting the complexity of microbial colonisation.

Although direct causal links between the milk microbiome of women with type 1 diabetes and infant gut colonisation remain to be established, evidence from related contexts supports a plausible connection. Studies in healthy mother–infant dyads demonstrate that milk-derived microbes, including *Streptococcus* and *Rothia*, colonise the infant gut and contribute to functional outputs such as short-chain fatty acids production [44], whereas gestational diabetes has been found to be associated with changes in breastmilk composition (human milk oligosaccharides and immunoglobulins) and the infant gut microbiome [15, 16]. Moreover, in infants at risk for type 1 diabetes, early-life gut microbiota deviations, including a decrease in bacteria producing short-chain fatty acids, have been observed before the onset of islet autoimmunity [45]. Together, our finding of delayed enrichment of key breastmilk taxa in mothers with type 1 diabetes, while not yet directly connected to infant outcomes, aligns with a biologically plausible link between altered early microbial exposure and downstream gut and immune development. Further studies are required to determine the impact on infant outcomes. Nevertheless, the findings underscore the importance of counselling women with type 1 diabetes to prepare for breastfeeding, as well as considering approaches such as maternal dietary modification, probiotic supplementation or microbial seeding that could promote early gut microbiome colonisation.

## Supplementary Information

Below is the link to the electronic supplementary material.

ESM (PDF 323 KB)

ESM Tables (XLSX 93 KB)

## Data Availability

Demultiplexed raw sequencing datasets supporting the conclusions of this study have been publicly available since 5 May 2026 and can be accessed through the NCBI Sequence Read Archive (www.ncbi.nlm.nih.gov/sra; BioProject PRJNA1356526). Data are available without access restrictions for secondary analyses and research use.

## Abbreviations

FDR: False discovery rate
GRS: Genetic risk score
OTU: Operational taxonomic unit
PCoA: Principal component analysis
sIgA: Secretory IgA

## Code availability

All R code used to perform statistical analyses has been publicly available since 18 April 2026 through GitHub (https://github.com/RothSchulze/Delayed-maturation-of-the-milk-microbiome-in-women-with-type-1-diabetes-.git). The repository includes QIIME2 command logs for sequence processing, R Markdown scripts and associated R objects containing taxonomic profiles and metadata used for statistical analyses. These resources are openly available without access restrictions.
