# Supplementary material for: Delayed maturation of the milk microbiome in women with type 1 diabetes
Source: Diabetologia. 2026 Jun 30;69(10):2833–46. doi: 10.1007/s00125-026-06791-6 (PMC13541889; doi:10.1007/s00125-026-06791-6)
Supplement: Supplementary file 1 [file 125_2026_6791_MOESM1_ESM.pdf]

Electronic Supplementary Material
Delayed maturation of the milk microbiome in women with type 1 diabetes

Alexandra J. Roth-Schulze[1], Katrina M. Ngui[1], Guinevere Martin[2], Pat Ashwood[2], Rebecca L. Thomson[2], Enrique Zozaya-Valdes[1], Yuan Gao[1], John M Wentworth[1,3], Maria E Craig[4], Anthony Hunyh[5], Jennifer J. Couper[2], Megan A. S. Penno[2] and Leonard C. Harrison[1], for the ENDIA Study Group.

1. Walter and Eliza Hall Institute of Medical Research, Melbourne, VIC 3052, Australia and Department of Medical Biology, University of Melbourne, Melbourne, VIC 3010, Australia.

2. Adelaide University, Robinson Research Institute, Adelaide Medical School, University of Adelaide, Adelaide, SA 5005, Australia.

3. Department of Diabetes and Endocrinology, Royal Melbourne Hospital, Melbourne, VIC 3050, Australia.

4. School of Women's and Children's Health, Faculty of Medicine, University of New South Wales, Sydney, NSW 2052, Australia and Institute of Endocrinology and Diabetes, The Children's Hospital at Westmead, Sydney, NSW 2145, Australia.

5. Faculty of Health, Medicine and Behavioural Science, The University of Queensland, Brisbane, Queensland, Australia and Department of Endocrinology & Diabetes, Queensland Children's Hospital, South Brisbane Queensland, Australia.

Corresponding author: Leonard C Harrison, Walter and Eliza Hall Institute of Medical Research, 1G Royal Parade, Parkville, 3052, VIC, Australia; harrison@wehi.edu.au ORCID 0000-0002-2500-48944

Abbreviations

Operational taxonomic unit (OTU)

PCoA Principal coordinates analysis

FDR False discovery rate

T1D Type 1 diabetes

BMI Body mass index

ESM Tables

ESM Table 1. Alpha diversity analysis of the milk microbiome.

ESM Table 2. Integrated genetic and metabolic alpha diversity analyses.

ESM Table 3. Indicators of delayed Lactogenesis II in women with and without T1D.

ESM Table 4. Beta diversity comparison for breast milk microbiomes.

ESM Table 5. Differential abundance analysis for breast milk microbiome.

ESM Table 6. Alpha diversity analysis of the infant gut microbiome

ESM Table 7. Beta diversity comparison for infant gut microbiomes.

ESM Table 8. Differential abundance analysis for infant gut microbiome.

ESM tables are provided separately in an accompanying supplementary Excel file.

Table of Contents

ESM Methods ... **3**

Study population ... 3

Milk sample collection and DNA extraction ... 4

DNA marker amplification and sequencing ... 4

Bacterial sequence analysis ... 5

Removal of technical contaminants and non-bacterial sequences ... 6

Alpha diversity analysis ... 7

Beta diversity analysis ... 9

Differential abundance analysis ... 10

Infant gut microbiome analysis ... 12

References ... **12**

ESM Figures ... **15**

ESM Figure 1. Breast milk microbiome taxonomic composition. ... 15

ESM Figure 2. Breast milk microbiome taxonomic composition. ... 15

ESM Figure 3. Breast milk microbiome composition by T1D status and timepoint. ... 16

ENDIA Study Group authors 2025 ... **16**

ESM Methods

Study population

As of the cut-off date (26th November 2018), the present study used all available milk samples collected between birth and 15 months postpartum as part of the Environmental Determinants of Islet Autoimmunity (ENDIA) pregnancy–birth cohort study [1]. Samples were analysed from 118 pregnancies, 69 of which were from women with T1D . The main criterion for participation in ENDIA was a child with a first-degree relative with T1D. Women who provided written informed consent were enrolled in the study between 2013 and 2019 at eight clinical sites in Australia. The study was approved nationally by the Women's and Children's Health Network Human Research Ethics Committee in Adelaide, acting as the lead under the Australian National Mutual Acceptance Scheme (reference number 2020/HRE01400). Conduct in Western Australia was also approved by the Child and Adolescent Health Service Human Research Ethics Committee (RGS0000002402). Governance approval was obtained at all participating sites. The investigators and study staff performed the study in accordance with International Conference on Harmonisation Good Clinical Practice guidelines, the Declaration of Helsinki, the Australian National Statement on Ethical Conduct in Human Research, and the Australian Code for the Responsible Conduct of Research. All participants provided written informed consent and were free to withdraw from the study at any time. Women self-reported their ethnicity. Demographic, pregnancy and birth data were collected prospectively, and clinical data were verified retrospectively against medical records.

Milk sample collection and DNA extraction

Women self-collected milk samples using a sterile 1 ml syringe (colostrum) or sterile 70 ml flat bottom screw cap container (breastmilk) at seven timepoints: B1= 0-3 days postpartum (a proxy for Lactogenesis Stage I or colostrum), B2= 4-10 days (a proxy for Lactogenesis Stage II), V1= 3 months, V2= 6 months, V3= 9 months, V4 = 12 months and V5 = 15 months (the last five timepoints being proxies for Lactogenesis Stage III). Milk was centrifuged at 10,000xg for 10 minutes at 4°C and DNA extracted from the pellet with a Maxwell RSC Purefood and Pathogen Kit (Promega). DNA concentration was determined fluorometrically with an Invitrogen Qubit dsDNA Assay kit (ThermoFisher Scientific), and purity was assessed based on 260/280 nm and 260/230 nm absorbance ratios determined in a DeNovix DS-11 Spectrophotometer (DeNovix Inc.). DNA integrity was checked by agarose gel electrophoresis. Samples were stored at −20°C until sequencing.

DNA marker amplification and sequencing

The V4 region of the 16S rRNA gene was amplified using primers 515F/806R for bacterial analysis [2]. Each sample was amplified in duplicate by PCR and the PCR products were visualised by agarose gel electrophoresis and quantified using Agilent 4200 TapeStation System. Equimolar amounts of purified amplicons were pooled and both PCR replicates were independently sequenced to ensure sufficient DNA yield using the Illumina MiSeq platform with version 3 600 cycle (2×300 bp) kit. Samples were sequenced in three different sequencing runs (runs 11, 16 and 22). Run 11 included samples from three water controls (WC; PCR-grade water to detect reagent contamination), three no-template controls (NTC; PCR reactions without added DNA to monitor for contamination during amplification), and three kit blank controls (KBC; DNA extractions performed without sample material to identify contamination introduced during extraction). Run 16 included two WC, three NTC and four KBC and run 22

included three WC, three NTC and five KBC. Each control sample was amplified and sequenced in duplicate.

Bacterial sequence analysis

To preserve the integrity of run-specific sequencing characteristics, each run was processed independently in QIIME2 (version 2024.10) [3] for demultiplexing, quality filtering, denoising using DADA2 ('qiime dada2 denoise-paired') [4] and taxonomic classification. This strategy was adopted to allow DADA2 to model the error rates unique to each sequencing run, thereby improving the accuracy of amplicon sequence variant (ASV) inference and minimizing artifacts due to run-specific quality differences. Independent processing also facilitated the assessment of technical controls and potential batch effects across runs.

From each run, feature tables and representative sequences were obtained and taxonomy was assigned using a naive Bayes classifier pre-trained on the SILVA 16S database (version 138, 99% similarity) [5]. The resulting ASV tables and taxonomic annotations were imported into R (version 4.4.2 2024-10-31) [6] using the 'phyloseq' package [7] v1.50.0. Technical replicates were merged within each run prior to integration. ASV tables, taxonomic profiles and associated metadata from all three runs were combined into a single phyloseq object (PO1).

To enable phylogeny-based analyses, representative sequences from all runs were merged in QIIME2 and aligned using MAFFT, followed by construction of an approximate maximum-likelihood tree with FastTree. The resulting phylogenetic tree was imported into R and added to PO1 to generate an updated phyloseq object (PO2). ASVs in PO2 were agglomerated into operational taxonomic units (OTUs) based on the phylogenetic tree using a co-phenetic distance threshold of 0.03 (tip_glom). OTUs contributing <0.01% of the total abundance were removed to reduce noise and focus on more meaningful signals. Only samples with more than 1,000 sequences were retained for the final analyses.

To standardise sequencing depth (ranging from approximately 1,000 to 83,000 reads), all samples were rarefied to 1,000 sequences without replacement using rarefy_even_depth. This threshold balanced sample retention with adequate depth to capture variation in alpha and beta diversity in a low biomass sample type. Normalised data were used for all statistical analyses, except for differential abundance analysis.

Removal of technical contaminants and non-bacterial sequences

To ensure that downstream analyses reflected true bacterial community composition, both technical and biological contaminants were filtered from phyloseq object PO2. First, taxa with uninformative or missing kingdom-level classifications (i.e., “Unk” or “Unassigned”) were excluded. To further remove non-bacterial sequences, taxa annotated as mitochondria or chloroplasts were filtered out based on their classification at the family, genus or class level, as these likely represented host-derived or plant-derived DNA. Samples with zero remaining reads after this step were removed.

In addition, 24 OTUs present in the negative control samples (kit blanks, water controls, and no-template controls) were used to identify potential contaminants introduced during sample processing and were removed from the entire dataset. Samples with fewer than 1,000 sequences were removed. After contaminant filtering, samples from different runs but with same ID (which included subject ID and sampled timepoint; *n*=1) were merged, and those with zero sequence counts (*n*= 5) were discarded, as these likely consisted solely of contaminant-associated taxa. This multistep filtering strategy was designed to remove both biological and technical contaminants, including host DNA, reagent-associated sequences, and spurious low-

confidence taxa, thereby improving the accuracy and reliability of subsequent microbial community analyses.

Alpha diversity analysis

Alpha diversity (diversity within microbial communities), determined as richness (total number of OTUs) and the Inverted Simpson (InvSimpson) index (a measure of the evenness of taxa distribution), was calculated using the function ‘estimate_richness’ from ‘phyloseq’. Differences in milk microbiome alpha diversity between women with and without T1D were assessed using generalized linear mixed models (GLMMs) with a negative binomial distribution (for observed richness) and a log link function (for InvSimpson index), as implemented in the glmmTMB package [8] v1.1.10 in R. This modeling framework was chosen to account for the overdispersion typical of count-based diversity metrics and the repeated measures structure of the study. Alpha diversity was specified as the outcome variable and maternal T1D status was treated as the main exposure.

Timepoint (a proxy for Lactogenesis Stage, as described above) was included as a fixed effect to capture longitudinal trends and an interaction term between T1D status and timepoint to evaluate group-specific trajectories. Additional covariates included delivery mode (categorized as elective Caesarean, emergency Caesarean, spontaneous vaginal or assisted vaginal), known to shape the milk microbiome composition [9, 10] and, in the case of Caesarean, its strong association with both maternal T1D and delayed lactogenesis II [11, 12]; BMI category (underweight [BMI < 18], normal [BMI > 18 and < 25], overweight [BMI > 25 and < 30] or obese [BMI > 30]), which influences milk microbiota diversity and composition [10, 13]; infant sex (male/female), which has been associated with differences in milk microbial profiles [14]; and parity (nulliparous/multiparous), which modifies breastmilk diversity and composition

[10]. Although maternal antibiotic exposure was initially considered for inclusion in the models due to its known impact on the milk microbiome [15], ultimately it was excluded because of the high prevalence of missing data for this variable. Breastfeeding method (direct breast vs pumped) can influence the milk microbiome, potentially via retrograde flow of infant saliva or milk stasis with direct feeding [10, 16]. However, in our study the availability of a milk sample at a given timepoint indicated active, direct breastfeeding, obviating the need to include breastfeeding method as a variable in the statistical models. Random intercepts were specified for sequencing run (to account for technical batch effects) and for subject ID (to account for within-subject correlation over time). The model fit was evaluated using residual diagnostics from the DHARMa package [17] v0.4.7 and model comparisons based on Akaike Information Criterion (AIC).

A parsimonious, biologically informed model was identified using AIC-based comparisons. The variables parity, infant sex and delivery mode (with ΔAIC values less than 2 when excluded individually) were retained in the final model due to their biological relevance and potential confounding effects. BMI category and interaction between BMI and T1D status were removed from the final model based on improved model fit (ΔAIC = –2.57 and -8.1, respectively), in keeping with their lack of added explanatory value.

After the detection of a significant interaction between T1D status and timepoint in the final model, pairwise comparisons of estimated marginal means (EMMs) were performed to assess group-specific temporal changes in alpha diversity. EMMs were calculated on the response scale using the emmeans package v1.11.1[18] in R, pairwise contrasts were extracted using the summary() function on the response scale and statistical inference was performed using Wald-type confidence intervals and FDR correction for multiple comparisons. P-values were

corrected for multiple comparisons with the Benjamini–Hochberg false discovery rate (FDR) method. Analyses focused on consecutive timepoint comparisons (e.g., B1 vs B2, B2 vs V1, etc.) within each T1D group, which capture developmental transitions in milk composition (from colostrum to mature milk). Between-group comparisons (T1D vs non-T1D) were also performed at each timepoint to detect differences in alpha diversity across lactation stages. Statistical significance was defined as FDR (adjusted p value) < 0.05.

To evaluate whether genetic susceptibility or glycaemic control contributed to milk microbiome diversity patterns, additional analyses were performed. Maternal T1D genetic risk score (GRS) [19] was determined within non-T1D mothers. Maternal HbA_{1c} was measured in T1D mothers. HLA-DR type [20] was included as a covariate in the primary longitudinal model. Associations with richness and inverse Simpson diversity were assessed using the same mixed-effects modelling framework described above.

Beta diversity analysis

Beta diversity was analysed to identify compositional shifts over time in women with and without T1D. Two sets of pairwise permutational multivariate analyses of variance (PERMANOVA) were performed using Bray–Curtis dissimilarities, calculated using ‘phyloseq’ (function distance, method = ‘bray’) on proportional log-transformed [i.e. base=exp(1)] data [i.e. log (x +1)] at the species, genus, family, order and phylum taxonomic levels.

Pairwise comparisons of microbial composition were made between consecutive lactation stages (B1 vs B2, B2 vs V1, …, V4 vs V5), stratified by T1D status. Separate PERMANOVA models were run for each group (T1D and non-T1D) and the following covariates were included: sequencing run, subject ID, pre-pregnancy BMI category, delivery mode, infant sex

and parity. The model structure was: Distance ~ (consecutive lactation stages) + Run + Subject ID + BMI + Parity + DeliveryMode + InfantSex.

Differences in microbial composition between T1D and non-T1D women were evaluated at each individual timepoint. For this analysis, the dataset was subsetted by timepoint, and the same covariates as above were included in the model. Subject-level blocking was not necessary since the majority of women contributed only one sample per timepoint. These analyses were carried out in R using the vegan package (v2.7-1, accessed May 2025) [21]. Each test was based on 999 permutations. Results from all analyses were FDR-corrected (Benjamini–Hochberg). Significance was defined as FDR < 0.05.

Differential abundance analysis

To identify differences in specific taxa between the breastmilk of women with and without T1D, differential abundance was analysed in limma [22]. For this, taxa per sample tables with raw counts for each of the five taxonomic levels were employed. Only samples with more than 1000 sequences were included. Library sizes were normalized using the trimmed mean of log expression ratios with singleton pairing (TMMwsp) method [23] in edgeR, which is expected to perform better for data with a high proportion of zeros. Counts were transformed to log2-counts per million (logCPM), voom precision weights were calculated and limma linear models were fitted while allowing for loss of residual degrees of freedom due to exact zeros using the voomLmFit function [24, 25]. Here, “subject IDs” were considered as blocks to calculate the consensus correlation and account for multiple measurements (i.e., sample from the same woman, but different timepoint) while estimating contrast statistics using the contrasts.fit function and empirical Bayes moderated t statistics.

To build the model for the differential abundance analysis, the variables “T1D status” and “timepoint” were combined into a single factor and the comparisons of interest were defined as contrasts, e.g., for testing differences due to T1D status at B1 timepoint: T1DB1 = (NonT1D_B1 - T1D_B1) and for testing the difference from B1 to B2 timepoints within women with T1D: B1B2T1D = (T1D_B1 - (T1D_B2). Due to their known effect on the milk microbiome, tests included adjustments for parity, BMI, delivery mode and infant sex. In addition, the model design included an adjustment for sample sequencing batch. Adjusted group means were computed before performing the test: for factors, the contr.sum function was used, e.g., contrasts (SequencingBatch) <- contr.sum(levels (SequencingBatch). The mean abundance of each taxon in each group was computed by limma when fitting the model and are contained in the coefficient’s component (fit$coef). The standard errors are obtained as follows: fit$stdev.unscaled(,) * sqrt(fitc$s2.post). Note that after normalization the data are on the log2 scale. The Benjamini-Hochberg method was used to control the false discovery rate (FDR). FDR < 0.1, a common standard for statistical significance in exploratory analyses [26], was considered significant.

To improve the taxonomic resolution of OTUs found to be significantly differentially abundant, their representative sequences were aligned using BLASTn against the NCBI 16S ribosomal RNA database (accessed June 2025), excluding uncultured and environmental sequences [27]. Alignments were filtered to retain only those with a minimum identity of 98%. Where possible, ASVs were assigned to a unique species based on the top hit. In cases where multiple species shared identical alignment scores, all matching species were reported.

Infant gut microbiome analysis

All stool samples available from infants from women included in the study (*n*= 489) were analysed using the same 16S rRNA gene sequencing pipeline as for the breastmilk samples. Alpha diversity was calculated using standard richness and diversity metrics, and longitudinal differences between groups were evaluated using mixed-effects models. Beta diversity was assessed using Bray–Curtis dissimilarity and tested using PERMANOVA to evaluate compositional differences between timepoints and between infants born to mothers with and without T1D. Differential abundance analyses as described above were performed to identify OTUs that differed between groups at early timepoints.

References

1. Penno MAS, Couper JJ, Craig ME, et al (2013) Environmental determinants of islet autoimmunity (ENDIA): a pregnancy to early life cohort study in children at-risk of type 1 diabetes. BMC Pediatr 13:124. https://doi.org/10.1186/1471-2431-13-124

2. ENDIA Study Group*, Penington JS, Penno MAS, et al (2018) Influence of fecal collection conditions and 16S rRNA gene sequencing at two centers on human gut microbiota analysis. Sci Rep 8(1). https://doi.org/10.1038/s41598-018-22491-7

3. Bolyen E, Rideout JR, Dillon MR, et al (2019) Reproducible, interactive, scalable and extensible microbiome data science using QIIME 2. Nat Biotechnol 37(8):852–857. https://doi.org/10.1038/s41587-019-0209-9

4. Callahan BJ, McMurdie PJ, Rosen MJ, Han AW, Johnson AJA, Holmes SP (2016) DADA2: High-resolution sample inference from Illumina amplicon data. Nat Methods 13(7):581–583. https://doi.org/10.1038/nmeth.3869

5. Pruesse E, Quast C, Knittel K, et al (2007) SILVA: a comprehensive online resource for quality checked and aligned ribosomal RNA sequence data compatible with ARB. Nucleic Acids Res 35(21):7188–7196. https://doi.org/10.1093/nar/gkm864

6. Team (2021) RC (2021) R: A Language and Environment for Statistical Computing. R Foundation for Statistical Computing

7. McMurdie PJ, Holmes S (2013) phyloseq: An R Package for Reproducible Interactive Analysis and Graphics of Microbiome Census Data. PLOS ONE 8(4):e61217. https://doi.org/10.1371/journal.pone.0061217

8. Brooks ME, Kristensen K, Van Benthem KJ, et al (2017) glmmTMB balances speed and flexibility among packages for zero-inflated generalized linear mixed modeling

9. Cabrera-Rubio R, Collado MC, Laitinen K, Salminen S, Isolauri E, Mira A (2012) The human milk microbiome changes over lactation and is shaped by maternal weight and mode of delivery. Am J Clin Nutr 96(3):544–551. https://doi.org/10.3945/ajcn.112.037382

10. Moossavi S, Sepehri S, Robertson B, et al (2019) Composition and Variation of the Human Milk Microbiota Are Influenced by Maternal and Early-Life Factors. Cell Host Microbe 25(2):324-335.e4. https://doi.org/10.1016/j.chom.2019.01.011

11. Prior E, Santhakumaran S, Gale C, Philipps LH, Modi N, Hyde MJ (2012) Breastfeeding after cesarean delivery: a systematic review and meta-analysis of world literature123. Am J Clin Nutr 95(5):1113–1135. https://doi.org/10.3945/ajcn.111.030254

12. Neubauer S, Ferris A, Chase C, et al (1993) Delayed lactogenesis in women with insulin-dependent diabetes mellitus. Am J Clin Nutr 58(1):54–60. https://doi.org/10.1093/ajcn/58.1.54

13. LeMay-Nedjelski L, Butcher J, Ley SH, et al (2020) Examining the relationship between maternal body size, gestational glucose tolerance status, mode of delivery and ethnicity on human milk microbiota at three months post-partum. BMC Microbiol 20(1):219. https://doi.org/10.1186/s12866-020-01901-9

14. Williams JE, Carrothers JM, Lackey KA, et al (2017) Human Milk Microbial Community Structure Is Relatively Stable and Related to Variations in Macronutrient and Micronutrient Intakes in Healthy Lactating Women. J Nutr 147(9):1739–1748. https://doi.org/10.3945/jn.117.248864

15. Moossavi S, Azad MB (2020) Origins of human milk microbiota: new evidence and arising questions. Gut Microbes 12(1):1667722. https://doi.org/10.1080/19490976.2019.1667722

16. Patel SH, Vaidya YH, Patel RJ, Pandit RJ, Joshi CG, Kunjadiya AP (2017) Culture independent assessment of human milk microbial community in lactational mastitis. Sci Rep 7(1):7804. https://doi.org/10.1038/s41598-017-08451-7

17. Hartig F (2024) DHARMa: Residual Diagnostics for Hierarchical (Multi-Level/Mixed) Regression Models (p. 0.4. 7)[Dataset]. 10.32614/CRAN. package. DHARMa

18. Lenth R, Banfai B, Bolker B, et al (2025) emmeans: Estimated marginal means, aka least-squares means. R package version 1.11. 1. CRAN R project

19. Sharp SA, Rich SS, Wood AR, et al (2019) Development and Standardization of an Improved Type 1 Diabetes Genetic Risk Score for Use in Newborn Screening and Incident Diagnosis. Diabetes Care 42(2):200–207. https://doi.org/10.2337/dc18-1785

20. Nguyen C, Varney MD, Harrison LC, Morahan G (2013) Definition of high-risk type 1 diabetes HLA-DR and HLA-DQ types using only three single nucleotide polymorphisms. Diabetes 62(6):2135–2140. https://doi.org/10.2337/db12-1398

21. Oksanen J, Kindt R, Legendre P (2008) Vegan: Community ecology package.-R package version 2.7. 1 Pfrender, ME and Lynch, M. 2000. Quantitative genetic variation in Daphnia: Temporal changes in genetic architecture. Evolution 54:1502–1509

22. Ritchie ME, Phipson B, Wu D, et al (2015) limma powers differential expression analyses for RNA-sequencing and microarray studies. Nucleic Acids Res 43(7):e47–e47. https://doi.org/10.1093/nar/gkv007

23. Robinson MD, Oshlack A (2010) A scaling normalization method for differential expression analysis of RNA-seq data. Genome Biol 11(3):R25. https://doi.org/10.1186/gb-2010-11-3-r25

24. Law CW, Chen Y, Shi W, Smyth GK (2014) voom: precision weights unlock linear model analysis tools for RNA-seq read counts. Genome Biol 15(2):R29. https://doi.org/10.1186/gb-2014-15-2-r29

25. Lun ATL, Smyth GK (2017) No counts, no variance: allowing for loss of degrees of freedom when assessing biological variability from RNA-seq data. Stat Appl Genet Mol Biol 16(2):83–93. https://doi.org/10.1515/sagmb-2017-0010

26. Callahan BJ, DiGiulio DB, Goltsman DSA, et al (2017) Replication and refinement of a vaginal microbial signature of preterm birth in two racially distinct cohorts of US women. Proc Natl Acad Sci 114(37):9966–9971. https://doi.org/10.1073/pnas.1705899114

27. Mortensen MS, Rasmussen MA, Stokholm J, et al (2021) Modeling transfer of vaginal microbiota from mother to infant in early life. eLife 10:e57051. https://doi.org/10.7554/eLife.57051

ESM Figures

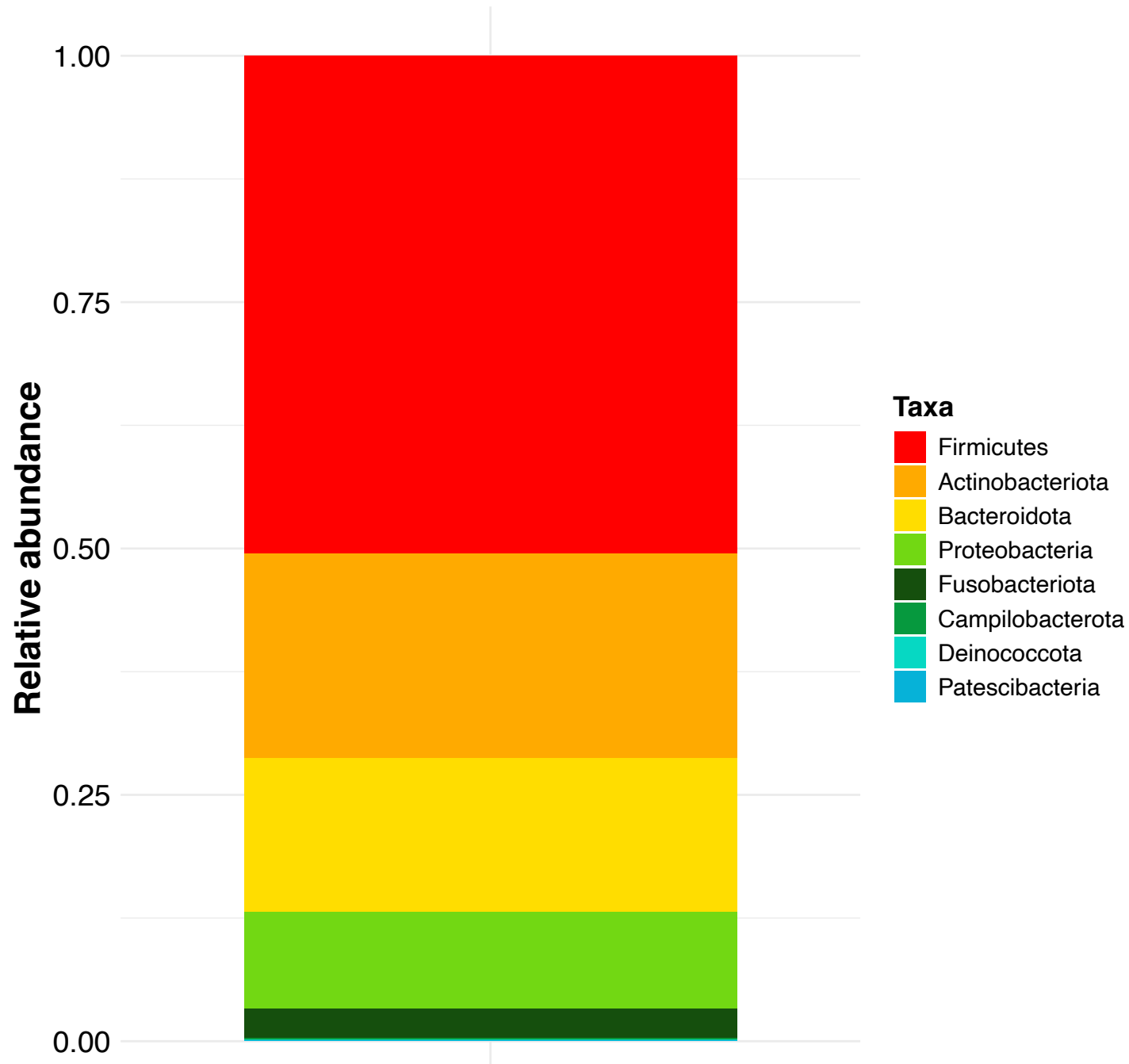

ESM Figure 1. Breast milk microbiome taxonomic composition.

Taxonomic composition of seven bacterial phyla found in breast milk samples collected from all women (with and without T1D) at seven timepoints postpartum.

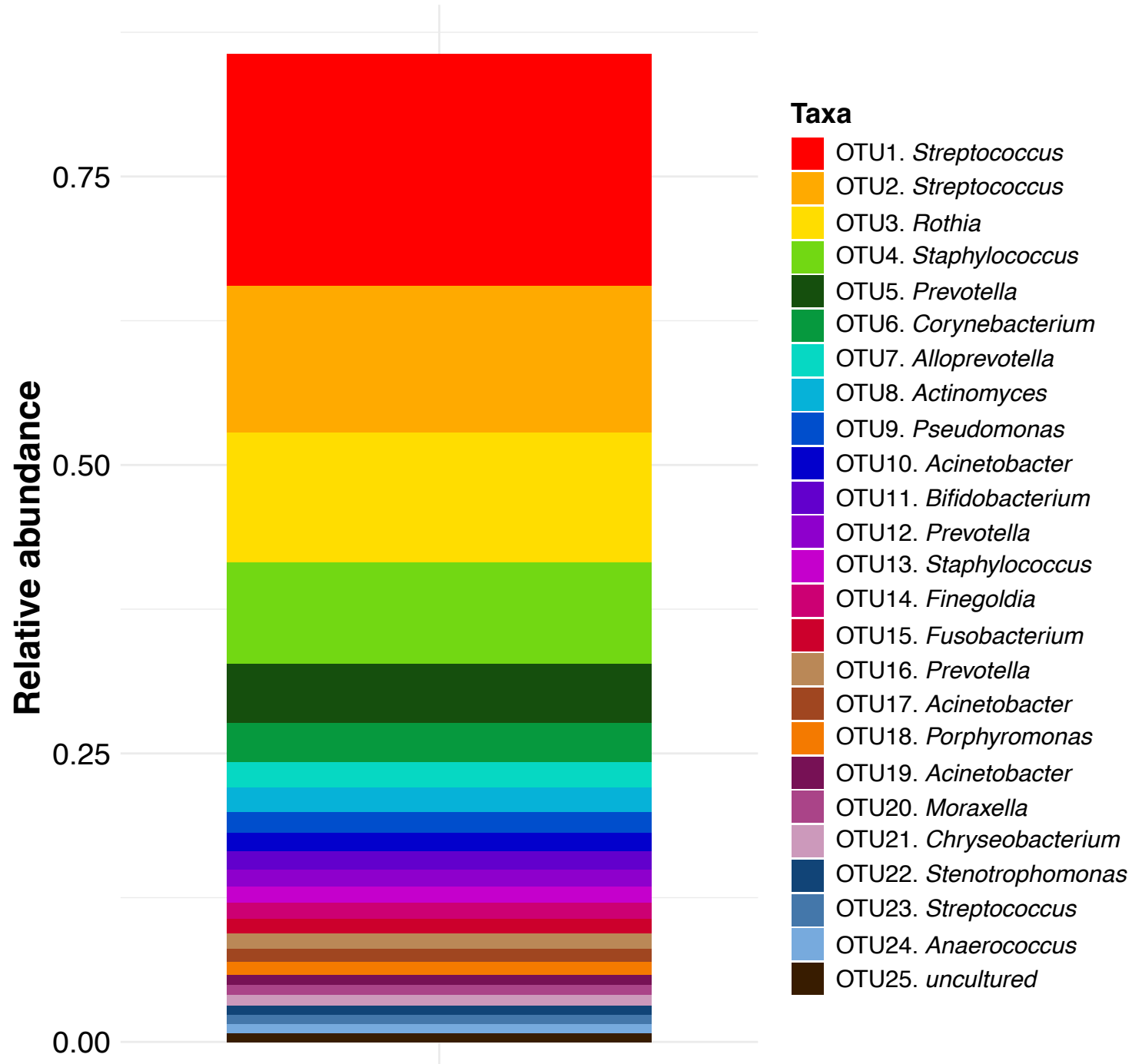

ESM Figure 2. Breast milk microbiome taxonomic composition.

Taxonomic composition of the 25 most abundant operational taxonomic units (OTUs) in breast milk samples collected from all women (with and without T1D) at seven timepoints postpartum.

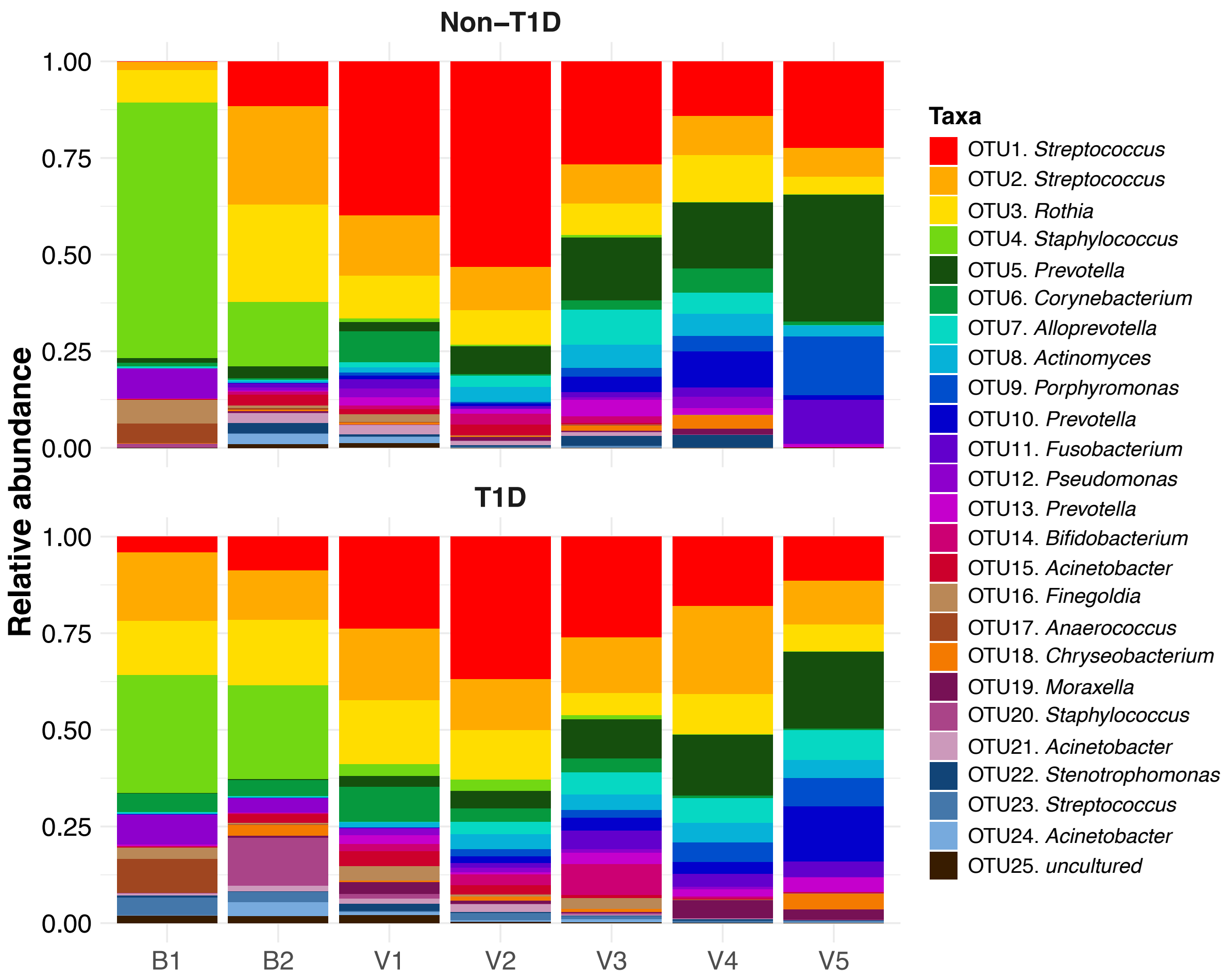

ESM Figure 3. Breast milk microbiome composition by T1D status and timepoint.

Taxonomic composition of the 25 most abundant operational taxonomic units (OTUs) in breast milk samples collected from women by type 1 diabetes status and timepoint.

ENDIA Study Group authors 2025

The ENDIA Study Group Consortium comprises Jennifer J. Couper, Leonard C. Harrison, Megan A. S. Penno, Maria E. Craig, Emma E. Hamilton-Williams, Rebecca L. Thomson, Jason Tye-Din, .Elizabeth A. Davis, Tony Huynh, Georgia Soldatos, John M. Wentworth, Peter Vuillermin, Patricia Ashwood, Simon C. Barry, Peter G. Colman, William D. Rawlinson, Aveni Haynes, Ki Wook Kim, Kelly J. McGorm, Grant Morahan.
